# Beyond Individual Triage: Regional Allocation of Life-Saving Resources such as Ventilators in Public Health Emergencies

**DOI:** 10.1007/s10728-020-00427-5

**Published:** 2021-02-06

**Authors:** Jonathan Pugh, Dominic Wilkinson, Cesar Palacios-Gonzalez, Julian Savulescu

**Affiliations:** 1 Faculty of Philosophy, Oxford Uehiro Centre for Practical Ethics, University of Oxford, Suite 8, Littlegate House, St Ebbes Street, OX1 1PT Oxford, UK; 2 John Radcliffe Hospital, Oxford, UK; 3 Murdoch Children’s Research Institute, Melbourne, Australia

**Keywords:** Resource allocation, Public health, Emergency, Coronavirus, Pandemic, Ventilators

## Abstract

In the first wave of the COVID-19 pandemic, healthcare workers in some countries were forced to make distressing triaging decisions about which individual patients should receive potentially life-saving treatment. Much of the ethical discussion prompted by the pandemic has concerned which moral principles should ground our response to these individual triage questions. In this paper we aim to broaden the scope of this discussion by considering the ethics of broader structural allocation decisions raised by the COVID-19 pandemic. More specifically, we consider how nations ought to distribute a scarce life-saving resource across healthcare regions in a public health emergency, particularly in view of regional differences in projected need and existing capacity. We call this the regional triage question. Using the case study of ventilators in the COVID-19 pandemic, we show how the moral frameworks that we might adopt in response to individual triage decisions do not translate straightforwardly to this regional-level triage question. Having outlined what we take to be a plausible egalitarian approach to the regional triage question, we go on to propose a novel way of operationalising the ‘save the most lives’ principle in this context. We claim that the latter principle ought to take some precedence in the regional triage question, but also note important limitations to the extent of the influence that it should have in regional allocation decisions.

## Introduction

The capacity of a healthcare system to care for those affected by a public health emergency will often depend on its access to scarce life-saving resources. Access to ventilators in the COVID-19 pandemic is one example of this phenomenon. Although these machines do not guarantee survival, they are a crucial piece of equipment in the present pandemic, as they are able to support patients with severe hypoxic respiratory failure. Unfortunately, in the first phase of the pandemic it appeared that the number of ventilators in many healthcare systems across the world was not enough to accommodate the number of patients likely to require them [4, 24, 29]. This was particularly so in the developing world, where critical care facilities are under-resourced [8, 14]. As such, healthcare workers in a number of countries were forced to make triaging decisions about which individual patients should receive this potentially life-saving resource. Call this the ‘individual triage question’.

The individual triage decisions that healthcare workers have faced were distressing, and it is appropriate that much of the ethical discussion prompted by the first wave of the pandemic has concerned which moral principles should ground these decisions. Whilst recognising the importance of that topic, in this paper we aim to address the ethics of broader structural allocation decisions in a public health emergency. Here we move from questions about allocating scarce medical resources between patients, to questions about allocating scarce medical resources across health care regions. Call this the ‘regional triage question’.

*The regional triage question:* How should a scarce healthcare resource be distributed between different health care regions?

To illustrate the problem more concretely, suppose that a second severe surge of coronavirus occurs in the winter of 2020/2021. Intensive care units in country X are under extraordinary pressure, and there is significant geographical variation in cases and projected peak demand for ventilators given the demographics and population density of each region. A new delivery of 1000 ventilators has arrived, and government officials have to choose how many should be distributed to each of the four regions of the country (Table 1).

**Table 1:** Characteristics of four hypothetical health regions at the start of a pandemic

Region	Population	Critical care capacity	Projected peak demand	Excess demand
A—Capital	9,000,000	1000	2000	1000
B—Large deprived urban area	10,500,000	700	2500	1800
C—Rural Area	5,500,000	300	2200	1900
D—Affluent younger city	7,000,000	500	1000	500

How should we decide where to send the additional ventilators in this case?^1^ As we shall explain, there are a number of reasons why moral frameworks for the individual triage question do not transfer straightforwardly to the regional triage question. Nonetheless, we shall argue that it is possible to revise a framework that some of us have defended elsewhere for the individual triage question, so that it can be applied to the regional triage question.

It should be acknowledged that the issues we raise here are particularly salient at the beginning of a public health emergency, when nations may not have had time to source adequate supplies, where there is uncertainty about the likely trajectory of the emergency, and where there are regional differences in the severity of the emergency and existing capacity to respond.^2^ We concentrate on the allocation of ventilators, though it is worth noting that these may not be the rate limiting step in the provision of treatment in the current pandemic.^3^

However, the case study of ventilators in the coronavirus pandemic is an instance of a more general phenomenon. Similar ethical questions may arise with respect to other potentially life-saving resources in the COVID-19 pandemic,^4^ as well as other scarce resources in future public health emergencies, such as those that may be prompted by infectious disease or natural disasters.^5^ The principles that we describe may be relevant to other therapies in this and future public health emergencies.

Our analysis will focus on two competing ethical values for allocation—benefit (particularly understood as numbers of lives saved) and fairness (particularly in terms of equal access to treatment). Those are not the only ethical values that are relevant for allocation decisions, and there are a range of views about the different weight that these (and other values) should have in such decisions. Yet these values provide an important starting point for discussing the ethics of regional allocation, since they both derive support from a wide range of moral theories. Moreover, there are a number of important questions about how these particular values should be operationalised in the context of regional allocation decisions. These questions are our primary focus in this paper. We will consider towards the end of the paper the different ways in which these and other values might be taken into account in an overall model of ethical regional triage. To be clear though, our primary aim in this paper is is to argue for how we ought to operationalise two particularly salient values that ought to factor into any plausible ethical approach to regional allocation.

To begin, we provide a necessarily brief overview of the moral principles that are operative in individual triage decisions.

## Principles for Individual Triage

Whilst there have been a number of accounts of ethical resource allocation in the COVID-19 pandemic, in the interests of clarity, we shall focus on a model outlined by Savulescu et al. [25].^6^ These authors note that in normal circumstances in which there are sufficient resources to meet clinical needs, then a principle of equal treatment should apply. This may recommend allocating additional resources on a first come, first served basis. Once local resources are consumed, later arriving patients should be transferred to other facilities to ensure access to treatment. However, in the context of scarce resources, this egalitarian principle would not be conducive to saving the most lives. It would mean, for example, that patients with a higher chance of survival arriving later in the course of a pandemic may not be able to be treated (if transfer is not possible or is too risky). Furthermore, egalitarianism as operationalized by a first come first served decision procedure will be unfair because it both unjustly affects populations that live far away from healthcare facilities, and it violates the ‘principle of temporal neutrality’.^7^ According to this principle, the time at which a harm occurs should not make a moral difference to the significance of that harm.

Savulescu et al. [25] therefore argue that when having to decide about the allocation of a scarce life-saving resource, the first level principle for such allocation should be to maximize the number of lives saved, when all other things are equal. They call this the ‘moral requirement to save the greatest number’ principle, suggesting that this should be a universal requirement of rationing, as it is supported by a diverse range of moral theories [12, 22].

*Moral requirement to save the greatest number principle* – When all other things are equal, allocate a scarce life-saving resource in a manner that will save more lives rather than fewer

In deciding how we can save the greatest number of lives when allocating a scarce life-saving resource Savulescu et al. [25] argue that we should allocate in accordance with each patient’s individual Resource Adjusted Probability Ratio (RAPR_i_). The RAPR_i_ takes into account a given patient’s probability of survival, and their expected level of resource demand. It is calculated as follows:

$$\begin{aligned} RAPR_{i} = \frac{Probability\; of\; survival}{Expected\; Resource \;Demand\;(Estimated\; duration\; of\; treatment/Average\; duration \;of\; treament)/}  \end{aligned}$$

The higher a patient’s probability of survival, and the lower their relative duration of treatment, the higher their RAPR_i_. Probability of survival can be calculated by a number of prognostic factors, including biological age, frailty and comorbidity.^8^ For example, if a patient has a 60% chance of survival and the predicted length of stay is 10 days, whereas the average length of stay is 5 days, the patient’s RAPR_i_ is 30%. The RAPR_i_ can be used to classify patients into categories for the purposes of individual triage decisions. For instance, a healthcare system may set a certain threshold, such that only patients scoring above it would be candidates to receive scarce life-saving resources.

However, there may be circumstances in which we have to invoke principles beyond the save the most lives principle. For instance, there may not be enough ventilators to supply to all patients over a given RAPR_i_ threshold.^9^ In such a scenario, Savulescu et al. [25] claim that we must invoke further allocation principles. However, unlike the ‘moral requirement to save the greatest number’ (which should be understood as a universal requirement of rationing), the further moral principle(s) that one may choose to invoke to decide cases of individual triage (once the save the most lives principle has been applied), will depend to a considerable extent on the values that the society and healthcare system in question chooses to prioritise. For instance, one might invoke an egalitarian principle that affords all individuals above the RAPR_i_ threshold an equal chance of receiving a ventilator. Second, one might make a second triage in accordance with a utilitarian principle, by basing allocation within this group on expected length or quality of life. Third, we might prioritise certain groups such as healthcare workers and/or younger patients on either desert-based or utilitarian grounds. Fourth, we might offer those with moderate chances of survival the opportunity for a fixed duration trial of treatment followed by withdrawal (if the patient further deteriorates or if significant improvement does not occur).

## Principles for Regional Triage

A number of countries have faced (or are facing) the regional triage question in reality. For instance, in the UK, a report in the Health Service Journal suggests that NHS trust chiefs were told in March 2020 by NHS England that additional ventilators will be deployed to “areas with the most immediate need, on a fair share basis relative to patient ventilation need” [5]. If ventilators were deployed to areas in the UK with the most *immediate* need, then there would likely be significant inter-regional inequality with regards to both the number of additional ventilators allocated to each region, and their post-allocation critical care capacity. Indeed, the report suggested that London would likely be prioritised in the first wave under the apparent NHS England approach, and that some medical professionals had thus called for the adoption of an alternative per capita approach to allocation [5].

To simplify our investigation of these principles, and because of limited publicly available data, we shall return to our hypothetical example to guide our philosophical assessment of potential regional distribution principles.

### Egalitarianism

In the individual triage question, the most straightforward egalitarian procedure is to allocate on a first come first serve basis or via a lottery; applying these procedures can mean that any patient has an equal chance of receiving care. The first thing to note in the regional triage question is that a first come first served approach will very seldom be functionally equivalent to a lottery. Allocating on the former basis is only equivalent to a lottery on the assumption that each region has an equal chance of ‘being first in the queue’, of needing the scarce resource before others.^10^ However, it is unlikely that different regions will all have an equal chance of being ‘first in the queue’ in this sense, given demographic variation between regions and different levels of population density.

With a lottery allocation, we could expect each region to receive the same number of additional ventilators. Although a lottery would ensure that each region had an equal chance of receiving additional ventilators, it not clear that this is the sort of equality that should really matter for the egalitarian. What should really matter for egalitarians is rather whether individuals in different regions have equal access to care; does an individual in region A have the same chance of accessing a ventilator as an individual in region B? However, if that is the case, then egalitarianism lends greater support to a regional allocation of ventilators on a *per capita* basis (rather than a lottery), as illustrated in Table 2.

**Table 2:** Simple egalitarian allocation on pure per capita basis

Region	Population	Rounded Proportion of Additional Ventilators on a per capita basis (%)
B	10,500,000	33
A	9,000,000	28
D	7,000,000	22
C	5,500,000	17

Yet, even this simple per capita approach does not capture all that should matter for the egalitarian; it fails to acknowledge that regions do not have equal *existing* per capita critical care capacity. Accordingly, if the aim of additional ventilator allocation is to (solely) ensure that allocation enables equal treatment access across regions, then allocation should be performed on the basis of the projected per capita demand of each region, in *excess* of their current capacity. As the table below illustrates, this approach generates a quite different prioritisation of regions (Table 3).

**Table 3:** Modified egalitarian allocation on projected excess per capita basis

Region	Population	Existing critical care capacity (number of existing entilator equipped beds)	Projected peak demand	Excess demand	Excess demand per capita (number of additional beds needed per 100’000 people)	Rounded proportion of additional ventilators on projected excess per capita basis (%)
C	5,500,000	300	2200	1900	34.5	49
B	10,500,000	700	2500	1800	17.1	25
A	9,000,000	1000	2000	1000	11.1	16
D	7,000,000	500	1000	500	7.1	10

We suggest that allocating on the basis of projected per capita *excess* demand would constitute the most plausible egalitarian approach to allocation. To illustrate how different approaches may roughly play out on the level of individual cities, estimates suggest that Bristol and Liverpool in the UK have a comparable population size (roughly between 450'000 and 500,000) [2, 16], and roughly comparable levels of adult critical care capacity (according to NHS statistics from January 2020) [19].^11^ Yet, at the start of May 2020 (when this paper was initially written), Liverpool had more than twice the number of cases of COVID-19 as Bristol (292 vs 125 cases per 100,000) [6]. Suppose we had to decide where to allocate additional ventilators on this data. The *simple* prospective egalitarian allocation based on a purely per capita basis would send equal numbers of ventilators to Bristol and Liverpool. We suggest that this would have been a profound mistake. The data outlined above suggests that the latter might have twice the excess per capita demand of the former. A more plausible modified egalitarian allocation at this point would thus potentially send more ventilators to Liverpool.

However, this answer, too, might be problematic. Depending on other variables that we analyse below, such an allocation may mean that fewer lives are saved.

### The Save The Most Lives Principle

Perhaps paradoxically, a policy of prioritising immediate need in the regional distribution of additional ventilators may be one way of operationalising the save the most lives principle. *Prima facie,* such a policy might appear to be a version of the egalitarian ‘first-come first served’ approach; it may entail allocating extra ventilators first to those areas of the country affected most severely earliest in the pandemic—potentially at the expense of those affected later. We shall now explain why regional prioritisation according to immediate need may be at least partly justified, but that we must also factor in other considerations in order to fully operationalise the save the most lives principle.

For individual triage, we noted that priority may be given to individual patients based on the RAPR_i_. This essentially helps us to estimate how much good a ventilator is likely to achieve for an individual patient in the numerator (assessed in terms of that individual’s probability of survival) and how efficiently it is likely to achieve that good in the denominator (assessed in terms of that patient’s relative expected resource demand). How can we similarly inform decisions about how to save the greatest number of lives via the regional distribution of additional ventilators?

#### The Numerator: How much good will additional ventilators achieve?

Consider first how much good additional ventilators will achieve. If our main concern is to save the most lives possible, then the first relevant assessment of regional need should concern the raw number of projected regional cases. If we are interested in maximizing the number of lives we save, then a region that is expected to have a higher number of individuals requiring ventilation has a greater need than a region with a lower number, even if that lower number nonetheless constitutes a higher proportion of its population.^12^ In turn, regional projected need can be modelled on the basis of population variables and the number of confirmed cases.^13^

However, the first point of complication here is that assessing a region’s demand is a more complex matter than assessing a presenting patient’s probability of survival in the individual triage question. Such assessments will often make projections about how the public health emergency may progress, and such projections must unavoidably rely on various assumptions.

This complication suggests one virtue of prioritising areas with the most immediate need. In our discussion of the individual triage question, we noted that the principle of temporal neutrality states that the time at which a harm occurs should not make a moral difference, other things being equal. However, this principle is compatible with the claim that we can have a greater degree of certainty at different points of time about whether or not a harm will occur. Moreover, this greater degree of certainty can make a moral difference about what we ought to do. Indeed, it may be that there is a predictable difference in projected need between regions that reach their ventilator capacity earlier or later in the course of the pandemic. If lockdown measures are effective in helping to ‘flatten the curve’, then those regions that are hit hard at an earlier stage of the outbreak may end up having a higher relative surge than those regions that are affected at a later stage in the outbreak. If that is right, we may save more lives by providing additional ventilators to those hospitals affected early, compared to those affected later.

The strength of our moral reason to prevent a harm is a function of both the magnitude of the harm, and its probability. Accordingly, for the reasons outlined above, we will often have stronger moral reasons to provide additional ventilators to a region that is further along its infection trajectory than the modelled projection of regions at earlier stages. Prioritising most immediate need thus need not violate the principle of temporal neutrality.

However, in considering the good that additional ventilators can do at a regional level, a further important question is whether there might be differences in regional survival rates. In our example, suppose that Region A has a lower demand for critical care than rural area Region C because the former has a younger population. However, the same factor might also mean that patients admitted to intensive care in Region A would have a higher chance of survival than those admitted to intensive care in the Region C. Imagine, for example, that 100 additional ventilators were made available for 1 week, enabling 100 extra patients to be admitted to intensive care. If those ventilators were provided to a region where the survival rate from intensive care was 50%, the allocation would result in 50 additional survivors. However, if those ventilators were provided to a different region where the patients were older/had a higher rate of co-morbidity, (and an average survival rate of 40%), only 40 lives would be saved.

That said, there are a number of difficulties with using assessments of regional survival rates to determine distribution. One difficulty is epistemic: it may not be possible to know what the relative survival rates are for different regions until it is too late to take that into account.

A second difficulty is more challenging—it is the relationship between demand, decision-making and outcome. For example, consider region X (Openshire) that decides not to restrict intensive care admissions, but attempts to provide intensive care to every patient with respiratory failure who would benefit, without any attempt to triage. Compare X with region Y (Closedbury), which anticipates a severe surge in demand, and decides to restrict admissions to intensive care to those patients with the highest chance of survival. Because Openshire has a less restrictive admission policy it will reach its capacity in terms of ventilators much sooner than Closedbury. It will also have a lower survival rate than Closedbury, since Openshire admitted patients who would have been excluded from intensive care in the other region. In this situation, it would appear to be problematic to provide extra ventilators preferentially to Closedbury, even if on paper they appear to have a higher survival rate.^14^

A third problem is more difficult still. It appears that there are differences in the rates of severe illness from COVID-19 between different ethnic groups [13, 21]. If that is correct, it might contribute to regional differences in survival rates following admission to intensive care. However, many will feel disquieted by the suggestion that this should be factored into a decision about regional ventilator allocation, even if it would mean saving more lives. Furthermore, data from the Office of National Statistics in the UK also suggests that COVID-19 is having a proportionally higher impact in more deprived areas [20]. Accordingly, underlying socio-economic inequalities may plausibly influence differences in regional survival rates. This potentially adds further issues regarding individual responsibility for illness in lifestyle diseases, like diabetes and hypertension. In any case, it may be doubly unjust to deprive those who already suffer from structural disadvantages from receiving ventilation.

There is one final factor that will significantly influence the good that additional ventilators will do in a given region. Ventilators are complex pieces of medical equipment that require trained staff; additional ventilators will not save more lives if they are sent to hospitals that do not have enough staff to operate them effectively. Accordingly, assessment of the good that additional ventilators will do must take into account the extent to which regions will be able to staff them.

#### The Denominator: How Efficient Would It Be To Send A Ventilator To A Given Region?

In the individual triage question, efficiency is largely a question of the amount of time that a particular patient will use a ventilator. The less time that the resource is needed for that patient, the more additional lives the resource can be used to save. It is less clear how that might be understood in terms of regional allocation. One possibility is that regions that have a shorter average intensive care length of stay might be prioritised over those with a longer length of stay. Providing 100 ventilators for a month to a region that has an average length of stay of 2 weeks (and 50% mortality) would save 100 lives. Providing the same number of ventilators to a region with an average length of stay of 1 week (and 50% mortality) could save 200 lives.

However, given the potential for regional differences in the time of peak ventilator demand, the efficiency of regional distribution may also be affected by the *timing* of sending additional ventilators to particular regions. In the first wave, Edge Health, an independent agency which advises NHS Trusts in the UK, developed a ‘pressure index’ which aimed to identify regions that are facing particularly severe pressure at a given time, calculated on the basis of reported deaths after controlling for demographics and pre-COVID-19 critical care capacity [7]. Such data could be used to form an estimate for the proportion of each region’s current critical care need that it would be able to address at a given time. Since it is likely that ventilators will become available progressively over time, it may be possible to allocate a tranche of new ventilators *now* to meet the needs of regions that are already reaching ventilator capacity, and then distribute a later tranche based on prevailing conditions when further ventilators become available (Table 4).

**Table 4:** Key considerations for the regional application of the ‘save the most lives principle

The Numerator—How Many Lives Will Additional Ventilators Save?	The Denominator—Efficiency of Distribution
*Projected Need*	*Average intensive care length of stay*
*Potentially affected by:*	Average ICU stay in the region, divided by the national average
Age	
Sex	
Regional Deprivation	
Ethnicity	
*Survival Rates*	*Current ICU Pressure*
*Potentially affected by:*	What proportion of patients requiring critical care can currently be accommodated?
Age	
Sex	
Regional Deprivation Ethnicity	Potentially informed by a ‘Pressure index’, calculated on the basis of reported deaths after controlling for demographics and critical care capacity
*Current Critical Care Capacity*	

One interesting question this raises is whether ventilators should be re-distributed. For example, suppose that the virus surges first in region C; 500 ventilators are made available to region C, and it is soon able to meet patient needs. Suppose now that there was then a surge in need in region D; should those ventilators be taken from region C and re-allocated? It would be hard to see why that shouldn’t occur, assuming the ventilators are not in use. If they were currently being used by patients, then this strategy of re-allocation would raise similar questions to the ethics of withdrawing treatment in the individual triage question [3, 17]. Some might feel that only spare ventilators should be redistributed to another region. However, if patients currently being treated in region C have a significantly worse prognosis than patients in region D (who will miss out if the ventilators are not redistributed), there is an ethical argument for mass reallocation. An alternative strategy in this scenario would be to redistribute patients to available supply by transferring them to hospitals with available capacity. Indeed, this has occurred in the international context, with Germany accepting patients from both Italy and France into their hospitals [1].

#### Operationalising The Save The Most Lives Principle For Regional Allocation Questions

The above considerations suggest that we should operationalise the save the most lives principle for regional allocation questions as follows. First, we must model the projected demand of each region *in excess of existing capacity*; doing so allows us to rank regions in terms of how many lives will likely be lost if critical care capacity does not increase. Recall that although an egalitarian approach would support a *per capita* assessment of need (which would adjust this figure for regional population), the save the most lives principle does not support such an approach.

In our example of country X, allocation purely on the basis of projected demand in excess of existing capacity would yield the following ranking of regional priority (Table 5).

**Table 5:** Allocation on the basis of Projected Excess Demand

Region	Population	Critical care capacity (Number of existing ventilator equipped beds)	Projected peak demand	Excess demand	Rounded proportion of additional ventilators on the basis of projected excess demand (%)
C	5,500,000	300	2200	1900	36
B	10,500,000	700	2500	1800	35
A	9,000,000	1000	2000	1000	19
D	7,000,000	500	1000	500	10

Allocating in accordance with the projected demand of each region in excess of existing capacity would be the most straightforward way to operationalise the save the most lives principle. However, it would also be incomplete, as it would fail to accommodate a number of important factors that will influence whether sending additional ventilators to a particular region will save the most lives. Rather than adjusting excess peak demand for population (as an egalitarian per capita approach requires), our discussion above suggests that the excess peak demand could instead be adjusted for survival rates, and efficiency of distribution.

To adjust for survival rates, one could calculate each region’s Adjusted Survival Rate (ASR) as follows:^15^

$$Total\;survivals/Total\;number\;of\;eligible\;patients\;treated = ASR$$

The ASR would aim to adjust for different admission criteria by calculating survival for a common cohort of patients—for example survival probability of patients aged 60–70 without severe co-morbidities.

Each region’s project peak excess demand could then be multiplied by its ASR to provide an estimate of how much good additional ventilators will do in that region. However, incorporating the regional ASR into this assessment introduces a number of controversial factors. Some regions may have lower ASRs as a result of having a large number of patients who are worse off, (for example, because they are more vulnerable to severe disease due to socio-economic deprivation or beacuse they are members of an at risk ethnic minority group). Others may have less effective critical care units, potentially as a result of past unjust allocation decisions.

The extent to which the regional ASR ought to feature in our assessment of how much good additional ventilators will do depends on whether we think it is acceptable for such inequalities to influence resource allocation decisions. We turn to this question in the next section. To conclude this part of the discussion though, we note that in order to operationalise the save the most lives principle in a comprehensive manner, we should also accommodate considerations of efficiency. Accordingly, excess projected need (potentially adjusted for regional ASR) could also be adjusted by each region’s average length of intensive care stay, and the current ICU pressure in the region. The former can be understood in terms of the length of the average ICU stay in the region, divided by the national average; the lower the figure, the more efficient the ICU.^16^ The current ICU pressure in a region can be understood in terms of the proportion of the region’s *current* critical care need that can be addressed with existing capacity; the lower the proportion, the greater the ICU pressure. This yields the following rough formula that could be used to comprehensively operationalise the save the most lives principle in the regional triage question into a Regional Allocation Index (RAI):

*Regional Allocation Index*

$$\frac{{\left[ {{\mathbf{Expected}}\;{\mathbf{Benefit}}} \right] = {\text{ Projected}}\;{\text{excess}}\;{\text{demand}} \times {\text{ASR}}}}{{\left[ {{\mathbf{Expected}} \, {\mathbf{Efficiency}}} \right] = \, \left[ {{\text{average}}\;{\text{length}}\;{\text{of}}\;{\text{intensive}}\;{\text{care}}\;{\text{stay}} \times {\text{current}}\;{\text{ICU}}\;{\text{pressure}}} \right]}}$$

Finally, if distribution on the basis of the RAI is to ensure that allocation saves the most lives possible, the number of ventilators that we allocate to a region on the basis of its RAI would be subject to a limit determined by the region’s capacity to safely staff additional beds.

The RAI formula could be used to determine which regional distribution of an additional life-saving resource would save the most lives; the higher a region’s RAI, the more lives that will be saved by sending additional ventilators to that region. In principle, the formula could *also* be applied to support the redistribution of critical care resources that were in place prior to an emergency. In some cases, saving the most lives may require the redistribution of existing resources, and not merely the allocation of additional resources.

Of course, it might be argued that the save the most lives principle is not all that matters here, and that other moral reasons would speak against a redistributive policy. We shall now consider the weight the save the most lives principle should bear compared to other principles in the regional triage question.

## Triaging Policies: From Individual to Regional Triage

In the individual triage question, Savulescu et al. [25] suggest that an individual’s RAPR_i_ may be used to set a certain threshold, such that only patients above the threshold would be candidates to receive a scarce life-saving resource. Other moral principles come into play if (i) we must decide between individuals in the above threshold group, *or*, (ii) if these individuals have received the good in question, and we must decide between individuals in the below-threshold group.

Our decision about where to set the relevant threshold in the individual triage question is sensitive to contextual factors. In particular, it must be relative to the numbers of patients needing the resource and the availability of the resource at a time. If there is low demand and ample resource, it is possible to provide intensive care to patients with a low chance of survival/need for prolonged support (“Low RAPR_i_”), whereas if there is overwhelming demand, the threshold may need to be set at a high level.

The level that we set the threshold at incorporates an important value judgment—the higher we set the threshold, the more that our allocation decision is determined by the ‘save the most lives principle’, at the expense of other important moral values. It would be possible to afford greater salience to other moral values (such as equality) by stipulating a lower RAPR_i_ threshold. Indeed, given the data suggesting differences in probability of survival amongst different age groups, some have argued that allocation decisions guided solely by the ‘save the most lives principle’ would serve to reinforce existing inequalities and constitute unfair discrimination [11]. Nonetheless, allocating ventilators on the basis of probability of survival may be morally permissible, and indeed lawful in some jurisdictions, even if it may constitute indirect discrimination, as long as the strategy represents a proportionate means of achieving a legitimate aim [26].

Another feature that should inform our deliberation on this point is the degree of confidence we give to our assessment of an individual’s RAPR_i_. The greater our confidence in the RAPR_i_ that we calculate for individual patients, the easier it is to justify strictly applying RAPR_i_ thresholds. However, uncertainty could be factored into thresholds through assessment of expected benefit.

Our decisions regarding the regional triage question should be sensitive to similar factors. However, there is a significant challenging in translating probability thresholds to the regional triage question. In the individual triage question, the decision to allocate is binary—a patient is either admitted to the ICU or they are not. Yet, the regional triage question is not binary in this sense. The question here is not simply whether a region should receive a ventilator, but also *how many* ventilators. Accordingly, rather than aim to determine a binary threshold, the save the most lives principle is best operationalised by allocating additional ventilators primarily *in proportion* to each regions’ projected demand in excess of existing capacity, adjusted in the ways outlined at the end of the previous section.

One benefit of using a threshold in the individual triage question is that it enables a clear policy on the way in which moral considerations beyond the ‘save the most lives principle’ can influence allocation decisions. However, since regional allocation is performed on a scalar rather than binary basis in the regional triage question, it is less immediately clear how these other moral considerations should be factored into allocation decisions. This is particularly problematic, since it might be argued that there are a number of reasons why the ‘save the most lives’ principle should have less prevalence in the regional triage question. First, as we discussed above, assessing the benefit and efficiency of allocating ventilators to a region will be a more complex matter than assessing a presenting patient’s RAPR_i_ in the individual triage question; accordingly, we may have less confidence in our assessments of which allocation will save the most lives.

Furthermore, many of the moral considerations beyond ‘saving the most lives’ are arguably more acute in the regional triage question. First, even if it may be proportionate to permit indirect discrimination in assessing individual patients, it might be argued that allowing indirect discrimination in the determination of triage at the regional level would significantly increase the number of people it affects, in a manner that renders it disproportionate. It might also generate concern about a “post-code lottery” in access to treatment.^17^ Second, it might also be argued that certain inequalities at the regional level have a particular moral significance. For instance, it might be argued that regional differences in both current critical care capacity and regional survival rate constitute an egregious form of inequality, in so far as they may be a result of past unjust decisions that have led to socio-economic deprivation. Such inequality might have greater moral weight on views that afford moral significance to the manner in which inequalities were produced.^18^

In view of these concerns, there is scope for reasonable disagreement about the precise weight that the save the most lives principle ought to have in regional allocation decisions. This is a question that calls for societal and political discussion about the values that we as a society want to be reflected in how healthcare is provided in a public health emergency. Despite these concerns, our view is that, like in the individual triage question, the save the most lives principle should take a certain degree of precedence in regional allocation decisions, and for similar reasons.^19^ We should first identify what distribution of additional ventilators would save the most lives. We may choose to deviate from that distribution for the sake of equality or fairness or other ethical considerations. But we would do so in the knowledge that this comes at a cost to the number of lives saved.

Of course, if there is no significant difference in the projected excess demand in different regions (adjusted for survival rates and efficiency), other moral principles, such as random allocation, should clearly come into play. Moreover, one could acknowledge the salience of other moral considerations (and adopt the spirit of the RAPR_i_ approach) by stipulating that allocation on the basis of the ‘save the most lives principle’ is only operative under a certain threshold of projected excess demand (adjusted for survival rate and efficiency). The latter strategy would serve to ensure that regions may not be deprioritised on the basis of the save the most lives principle, unless they have a very low need, survival rate and/or poor efficiency of ventilator usage.

Above the threshold, regions may compete on an equal level, and other competing moral principles might need to be operationalized to decide where to allocate the ventilators. In section II, we illustrated how egalitarian principles could be operationalised in this context, by allocating on the basis of excess per capita need. However, it might be argued that the egalitarian principle we outlined above fails to acknowledge the fact that some *per capita* shortfalls are also the result of past unjust allocation decisions. It might be argued that we should prioritise regions that have been subject to such injustice over those whose shortfall is not attributable to previous unjust allocation decisions.

In some cases, it seems plausible that regions with the most significant per capita shortfalls of critical care capacity may also be worse off than other regions on a number of other morally relevant metrics, such as economic well-being. In such circumstances, egalitarian principles and prioritiarian (and utilitarian) principles would converge on the verdict that we ought to prioritise these regions in our resource allocation. However, if there were a case in which the principles diverged, then a prioritarian principle could potentially be invoked to support allocating a scarce resource to a region that is worse off in some morally significant sense over one that nonetheless has a greater *per capita* shortfall. The question of when to give priority to the worse off (at the cost of equal treatment and potentially at the cost of benefiting fewer people) is the core ethical dilemma for prioritarianism.

Finally, and perhaps most controversially, a utilitarian approach to justice would speak in favour of taking into account the quality and length (and not just the number) of lives saved by regional allocation decisions, by taking into account, inter alia, the likely amount of QALYs that a particular distribution would achieve.

## Conclusion

The gravity of the individual triage question has been clearly apparent to healthcare professionals in the COVID-19 pandemic. The moral significance of the regional triage question we have discussed here is perhaps more abstract; yet, in the current context, the moral stakes it raises are high. Our decision about whether to allocate additional ventilators on the basis of the ‘save the most lives principle’ or on the basis of other moral values could potentially have implications for the ability of thousands of critically ill people to access life-saving care. It is therefore crucial that our answer to the regional triage question is ethically rigorous and transparent. We have concentrated on ventilators, but an ethical approach to regional allocation has clear relevance to other scarce life-saving resources in the COVID-19 pandemic, not to mention the allocation of such resources in other widespread public health emergencies that we may face in the future.

In this paper, we have explained how one framework for answering the individual triage question can be revised and extended in order to answer the regional triage question for the provision of scarce treatment. This approach affords precedence to the save the most lives principle, which we have suggested should be understood to be a universal requirement of ethical rationing in this context. We have outlined a formula that can be used to comprehensively operationalise the principle in the regional context, which takes into account both the benefit achieved by a particular regional allocation (assessed in terms of projected demand in excess of existing capacity, adjusted survival rate, and capacity to accommodate extra units of treatment), and the efficiency of doing so. We suggested that this formula should be used to identify which regional distribution of treatment would save the most lives. Whether we are allocating ventilators or other scarce medical treatment we may choose to deviate from that distribution for the sake of equality or fairness or other ethical considerations, for instance by adopting a per capita approach above a certain threshold or by giving priority to disadvantaged groups. However, any deviation from the save the most lives principle in a public health crisis requires strong moral reasons, given the number of lives at stake.

## Footnotes

1 We have been influenced in designing this hypothetical by considering the characteristics of different regions in the UK and questions about where to allocate ventilators. However, the issues are not specific to the UK, and these hypothetical regions should not be taken to be representative of actual locations.

2 Obviously, if there are no differences between areas in the nature of the problem, there is no great difficulty in deciding how to allocate treatment. For example, if all areas are completely overwhelmed, any distribution of ventilators may have a similar effect.

3 Ventilators require skilled personnel to operate them (with sufficient personal protective equipment), sufficient oxygen to run them, medications (e.g. sedatives) to keep patients sedated etc. Anecdotally, in the first wave of the pandemic in the UK, there were significant shortfalls in all of those factors affecting or threatening patient care, even though ventilator numbers were sufficient. https://www.theguardian.com/world/2020/apr/07/high-demand-for-oxygen-risks-system-failure-nhs-england-warns, https://www.bbc.co.uk/news/newsbeat-52440641https://www.bbc.co.uk/news/health-52150861.

4 For example, they may arise with respect to other existing treatment resources, such as Extra-Corporeal-Membrane-Oxygenation (ECMO) machines. See [15] They may potentially arise with respect to treatments that are currently under investigations, such as Remdesivir [28] and intravenous immunoglobulin treatment or monoclonal antibodies.

5 Notably, it is less clear that our analysis translates to *preventative* public health interventions such as vaccinations.

6 See also [18, 24, 27].

7 Savulescu et al. [25]. See also [9].

8 The RAPR_i_ assumes that patients in need of the treatment will all die without the therapy (in this case ventilators). If there is a greater than zero chance of survival without treatment, the relevant “probability of survival” is the difference between the chance of surviving without a ventilator, and the chance of surviving if treated with a ventilator. (Obviously, it would be irrational to prioritise a patient who had a high chance of surviving without treatment, and whose chances would be changed little by access to a ventilator).

9 Alternatively, there may be surplus ventilators once all patients in the high RAPR_i_ group have been given access, and there may still be a large group falling below the threshold that would benefit from access to a ventilator.

10 For discussion, see [10].

11 NHS trusts in the city of Liverpool have a sum total of 95 adult critical care beds, whilst NHS trusts in Bristol have 101 [19].

12 While not relevant to saving the greatest number, per capita assessments may be of moral relevance in other ways. For instance, it might be argued that it would be *fairer* to prioritise regions that will have a higher proportion of their population requiring ventilation. We shall return to this point below.

13 For an example of such modelling in the UK context, see Edge Health, ‘How Many More Critical Care Beds Are Needed Regionally?’.

14 The relevant survival rate for regional triage should include all patients admitted to a hospital, not solely those admitted to intensive care. This would take into account the probability of survival without intensive care and reduce selection effects. See also earlier footnote 8.

15 Of course, a more complex case-mix adjusted survival rate could be calculated.

16 One factor that might influence length of stay for an intensive care unit will be whether clinicians would consider withdrawal of treatment for patients with relatively poor prognosis who have failed to wean off support. If intensive care units are prepared to withdraw treatment in order to provide it to other patients with better prognosis, [3, 17] that would potentially increase the efficiency of ventilator usage and increase the priority of the region for allocation.

17 Such concerns typically focus on arbitrary regional boundaries and differences in distribution of treatment. While regional triage and allocation of a scarce treatment along the lines described in this paper would generate some differences between regions, those differences would not be “arbitrary”—they would be on the basis of justifiable ethical principles.

18 See Parfit’s discussion of deontic egalitarianism in [23].

19 We have defended this claim in relation to individual triage in [25].
